# Assessing Pain among Chinese Elderly-Chinese Health and Retirement Longitudinal Study

**Published:** 2018-04

**Authors:** Tong YU, Jun MA, Yan JIANG, Jian LI, Yunlong Gen, Yufeng WEN, Wenjie SUN

**Affiliations:** 1. Dept. of Medical Insurance, School of Humanities and Management, Wannan Medical College, Wuhu, China; 2. Dept. of Epidemiology, School of Public Health, Wannan Medical College, Wuhu, China; 3. Dept. of Global Biostatistics and Data Science, School of Public Health and Tropical Medicine, Tulane University, New Orleans, USA; 4. Dept. of Epidemiology, School of Public Health and Tropical Medicine, Tulane University, New Orleans, USA; 5. Dept. of Global Environmental Health Sciences, School of Public Health and Tropical Medicine, Tulane University, New Orleans, USA

**Keywords:** Pain, Prevalence, Socioeconomic, Elderly, China

## Abstract

**Background:**

Body pain is an important issue among elderly. The objective of this study was to access the association between the socioeconomic status and pain among elderly Chinese.

**Methods:**

This nationally representative sample cohort study, China Health and Retirement Longitudinal Study (CHARLS), was conducted to estimate pain prevalence and risk factors from Jun 2011 to Mar 2012. Body pain was evaluated by the questionnaires. Logistic regression model was applied to estimate the odds ratio (OR) and 95% Confidence Interval (95% CI) of body pain to identify the potential risk factors.

**Results:**

The prevalence of pain was increased with age (*P*<0.05). For moderate pain vs. no pain, doing agriculture job (OR 1.17; 95 CI 1.05–1.31), living in urban (OR 0.80; 95 CI 0.72–0.90), having a health problem (OR 1.55; 95 CI 1.20–1.99) is associated with moderate pain. For severe pain vs. no pain, primary school education (OR 0.65; 95 CI 0.54–0.78), junior high school education (OR 0.48; 95 CI 0.39–0.59), having a physical disability (OR 2.71; 95 CI 2.18–3.37), never drinker (OR 0.74; 95 CI 0.60–0.91), environment of urban (OR 0.54; 95 CI 0.46–0.63), having a health problem (OR 2.03; 95 CI 1.45–2.83) are associated with severe pain.

**Conclusion:**

Socioeconomic variables such as education, occupation and health conditions are associated with both moderate severe pains.

## Introduction

“Pain management in the elderly has increasingly become problematic as the aged population grows” (1). Approximately 60 million people live with chronic pain around the world and 30%–40% of individuals in China live with chronic pain (2). With aging, the prevalence of body pain increased (3). In China, pain is an important public health concern since China’s population has been aging rapidly and the proportion of elderly people will continue to grow. However, pain has not been commonly regarded as a health problem; instead, it is thought of as symptoms of other diseases and is often neglected (2).

Pain also causes many problems such as depression, the inability to work and disrupts relationships (2). The combination of chronic pain and comorbidities of other psychological disorders cause disability worldwide (2). The biological mechanisms involved in feeling pain are agnogenic and complex.

Of note, there is an association between socioeconomic status and pain. People who live in poorer neighborhoods are reported to have a higher prevalence of pain (4). Low socioeconomic status was associated with increased prevalence of low back pain (5). However, the study addressed on socioeconomic factors’ role on body pain among the elderly is limited (6). Pain centers in China use medication like analgesics as an approach to treating chronic pain (7).

Socioeconomic conditions impact the level of pain experience. Hence, the objective of this study was to access the association between the socioeconomic status and pain among elderly Chinese.

## Methods

### Study design and Settings

The data was from China Health and Retirement Longitudinal Study (CHARLS) based on a longitudinal survey for Chinese aged 45 yr or above and their spouses. The methodology of the study was reported elsewhere (8). In brief, the national baseline survey of 18994 respondents was carried out between June 2011 and March 2012. The respondents were followed up every two years through a face-to-face interview. The physical measurements were taken and blood samples were collected once in every two follow-up periods. The baseline survey provided information on demographic characteristics, socioeconomic status, biomedical measurements and health status.

### Participants and Sampling

In the CHARLS, a four-stage, stratified cluster probability sampling design was used to select representative samples. The primary sampling unit (PSU) was based on the villages in rural areas (formally village-level divisions in China, serve as a fundamental organizational unit for its rural population) and neighborhoods in urban areas (generally used for the urban administrative division below the district level, encompass 2000 to 10000 families). At the first stage, all county-level units were stratified by region and rural/urban status. A random sample of 150 county-level units in 28 provinces was selected to represent the socioeconomic and geographic patterns. (In China, counties are defined as the third level of the administrative hierarchy, a level known as “county-level”). In the second stage, we selected three PSUs within each county-level unit. In the third stage, the households were selected in each PCU, constructed based on maps. A random sample of 24 households was selected among residents aged ≥45 yr. If a chosen person aged ≥45, he/she becomes the main respondent and interviewed his or her spouse. Those individuals who were under 45 yr of age were excluded from the study, which is consistent with previous studies on CHARLS (9).

### Ethics

The current study is a secondary analysis of the de-identified CHARLS public data. The Medical Ethics Committee of Wannan Medical College approved the study.

### Variables

The main outcome variable in questionnaire on pain was 5 levels (no pain, mild pain, moderate pain, severe pain and worst imaginable pain) (10). We categorized them into 3 levels (no pain, moderate pain or severe pain): moderate pain was a combination of mild pain and moderate pain while severe pain was a combination of severe pain and worst imaginable pain. The socioeconomic status variables included education, occupation, and monthly expenditure on food, referenced by following study (11). Education was categorized into “Illiterate”, “Liberate”, “Primary school” or “High School or above”. Occupation was described by the question whether the participants were involved in agricultural job for more than 10 d in the past year and then was categorized as “yes” or “no”. Food expenditure was categorized into four categories (≤66.8 Yuan), (>66.8 Yuan and ≤139.4 Yuan) (>139.4 Yuan and ≤290.1 Yuan) and (>290.1 Yuan). The disease variables were described by the question whether the individuals had any one of the listed health condition (hypertension, dyslipidemia, diabetes, cancer, lung, liver, heart, stroke, kidney, digestive, psychological, memory related disease, arthritis, and asthma) and then was categorized as “yes” or “no”. The influencing factors consisted of age, gender, physical disability, residence, marriage status, disease, smoking and drinking status. Age was categorized into seven categories (45–49, 50–54, 55–59, 60–64, 65–69, 70–74 and 75+). Marriage was categorized as married or not married. Smoking status was categorized as “smoked” or “never smoked”. Drinking status was categorized as “regular drinker”, “occasional drinker” or “former drinker”. Physical disability was categorized as “Disability” or “Non-disability”. The type of residence was categorized as “urban” or “rural”.

### Statistical Methods

Statistical analysis was carried out using SAS 9.3 (SAS Institute, Cary, NC, USA). The frequency description was used to analyze the demographic and behavior characteristics of the participants. Univariate analysis was conducted to identify significant variables based on a *P*-value <0.25, based on Type 3 analysis of effects. Once the variables were selected, multivariate analysis by manual backward elimination was used based on a *P-*value of <0.05 and confounding variables were tested based on a 20%–30% difference between the crude and adjusted coefficients. The OR and 95%CI were computed and presented in table format.

## Results

### Baseline characteristics

The respondents have contained 16006 observations with 11 variables (age, gender, education, physical disability, job type, environmental setting, food expenditure, disease, smoking, drinking, and marriage status) (Table 1).

**Table 1:** Baseline characteristics of China health and retirement longitudinal study

***Variable***	***Pain***		
	**No Pain (0)**	**Moderate Pain (1)**	**Severe Pain (2)**
Age (yr) // 45–49	1.735	628	163
50–54	1.760	724	207
55–59	1.974	766	303
60–64	1.882	778	336
65–69	1.256	554	221
70–74	842	371	158
75+	881	401	148
Gender			
Male	5.422	1725	523
Female	4.905	2.497	1.012
Marital status			
Married	8.699	3.416	1.182
Non-married	1.629	805	353
Smoking			
Smoked	4.305	1.457	502
Non-smoked	6.019	2.762	1.033
Drinking // Former drinker	1.036	442	187
Never drinker	5.720	2.540	953
Occasional drinker	589	248	73
Regular drinker	2.973	986	320
Disability			
Yes	476	305	175
No	9.854	3.916	1.360
Monthly Food Expenditure (Yuan)			
If ≤66.8	3.258	1359	615
If >66.8 and ≤139.4	2.200	945	363
If >139.4 and ≤290.1	2.496	990	281
If >290.1	2.376	928	277
Education level			
Illiterate	2.347	1230	630
Liberate	1.813	839	333
Primary school	2.275	899	288
High school or above	3.895	1.254	285
Residence // Rural	5.977	2.677	1.125
Urban	4.353	1.545	411
Occupation			
Agriculture	5.410	2.366	842
Non-agriculture	4.876	1.846	688
Disease			
Yes	245	145	74
No	10.085	4.077	1.462

### Univariate analysis of the risk factors for pain

The univariate analysis revealed the comparisons of age, gender, education, physical disability, job type, environmental setting, food expenditure, disease, smoking status, drinking status and marriage status between moderate pain and no pain groups or between severe pain and no pain groups (Table 2).

**Table 2:** Univariate analysis of the risk factors for pain

***Variable***	***1 (mild pain) vs 0 (no pain)***		***2 (severe pain) vs 0 (no pain)***	
Age (yr)	Estimate	*P*-value	Estimate	*P*-value
45–49	Reference			
50–54	0.1949	0.0878	0.2569	0.0515
55–59	0.1112	0.3215	0.5937	<.0001
60–64	0.2053	0.0455	0.7795	<.0001
65–69	0.2076	0.0680	0.6841	<.0001
70–74	0.2495	0.0382	0.7397	<.0001
75+	0.2235	0.0693	0.6410	<.0001
Gender				
Male	Reference			
Female	0.4540	<.0001	0.7816	<.0001
Marital status				
Non-married	Reference			
Married	−0.2339	0.0019	−0.04423	0.0019
Smoking				
Non-smoker	Reference			
Smoker	−0.2302	0.0001	0.3624	<.0001
Type 3		<.0001		<.0001
Drinking				
Former drinker	Reference			
Never drinker	0.0106	0.8959	−0.0814	0.4314
Regular drinker	−0.2732	0.0047	−0.5327	<.0001
Occasional drinker	0.1167	0.0024	−0.4967	0.0024
Disability				
No	Reference			
Yes	0.4748	<.0001	1.0588	<.0001
Food expenditure (Yuan)				
If ≤66.8	Reference			
If >66.8 and ≤139.4	0.0896	0.1672	−0.0100	0.9039
If >139.4 and ≤290.1	−0.00136	0.9841	−0.4190	<.0001
If >290.1	−0.0308	0.7184	0.5350	<.0001
Education level				
Illiterate	Reference			
Primary	−0.3730	<.0001	−0.8404	<.0001
High School or above	−0.4526	<.0001	−1.3549	<.0001
Liberate	−0.1314	0.0477	−0.3308	0.0002
Residence				
Rural	Reference			
Urban	−0.3156	<.0001	−0.8401	<.0001
Occupation				
Non-agriculture	Reference			
Agriculture	0.2206	<.0001	0.2956	<.0001
Disease				
No	Reference			
Yes	0.4738	0.0002	0.8214	<.0001

### Multivariable analysis of the risk factors for pain

Table 3 shows the results of multivariate analysis of the association of moderate pain or severe pain with influencing factors. For moderate pain vs. no pain, female gender (OR 1.71; 95 CI 1.48–1.97), having a physical disability (OR 1.62; 95 CI 1.37–1.93), agriculture job (OR 1.17; 95 CI 1.05–1.31), environment of urban (OR 0.80; 95 CI 0.72–0.90), having a health problem (OR 1.55; 95 CI 1.20–1.99) is associated with moderate pain. For severe pain vs. no pain, age group of 55–59 (OR 1.66; 95 CI 1.30–2.11), age group of 60–64 (OR 1.74; 95 CI 1.37–2.21), age group of 65–69 (OR 1.59; 95 CI 1.23–2.06), age group of 70–74 (OR 1.56; 95 CI 1.17–2.08), female (OR 2.38; 95 CI 1.95–2.91), primary school education (OR 0.65; 95 CI 0.54–0.78), junior high school education (OR 0.48; 95 CI 0.39–0.59), having a physical disability (OR 2.71; 95 CI 2.18–3.37), never drinker (OR 0.74; 95 CI 0.60–0.91), environment of urban (OR 0.54; 95 CI 0.46–0.63), having a health problem (OR 2.03; 95 CI 1.45–2.83) are associated with severe pain.

**Table 3:** Multivariable analysis of the risk factors for pain

	***Moderate pain vs. no pain***			***Severe pain vs. no pain***		
**Variable**						
Age (yr)	**OR**	**95% CI**		**OR**	**95% CI**	
45–49	Reference					
50–54	1.208	0.959	1.523	1.316	1.016	1.704
55–59	1.105	0.885	1.381	1.657^*^	1.298	2.114
60–64	1.165	0.951	1.427	1.737^*^	1.367	2.207
65–69	1.188	0.951	1.484	1.590^*^	1.227	2.062
70–74	1.243	0.974	1.587	1.563^*^	1.173	2.082
75+	1.202	0.929	1.556	1.241	0.907	1.699
Gender						
Male						
Female	1.708^*^	1.477	1.974	2.380^*^	1.948	2.909
Education						
Illiterate						
Primary	0.854	0.736	0.992	0.647^*^	0.535	0.783
High School or above	0.866	0.749	1.002	0.481^*^	0.394	0.588
Liberate	1.006	0.878	1.152	0.900	0.753	1.074
Disability						
No						
Yes	1.623^*^	1.366	1.928	2.706^*^	2.175	3.366
Occupation						
Non-agriculture						
Agriculture	1.173^*^	1.052	1.308	0.966	0.837	1.115
Marital status						
Non-married						
Married	0.847	0.726	0.989	0.814	0.686	0.966
Drinking						
Former drinker						
Never drinker	0.866	0.738	1.015	0.735^*^	0.595	0.907
Occasional drinker	1.196	0.883	1.618	0.786	0.566	1.092
Regular drinker	0.859	0.714	1.033	0.787	0.624	0.992
Residence						
Rural						
Urban	0.803^*^	0.720	0.897	0.539^*^	0.459	0.633
Food Expenditure (Yuan)						
>66.8 and ≤139.4 vs. ≤66.8	1.100	0.966	1.252	1.015	0.861	1.197
>139.4 and ≤290.1 vs. ≤66.8	1.076	0.935	1.239	0.798	0.664	0.958
>290.1 vs. ≤66.8	1.140	0.959	1.356	0.854	0.705	1.034
Healthy						
Yes						
No	1.545^*^	1.202	1.987	2.026^*^	1.452	2.827
Smoking						
Non-smoker						
Smoker	1.104	0.960	1.269	1.201	0.995	1.449

Notes:

* *P*<0.05

## Discussion

Our results suggest that 35.9 % of the Chinese elderly suffers from chronic body pain. Moreover, socioeconomic variables such as education and occupation are associated with moderate and severe pain.

The prevalence of pain among the elderly varied from 15.2% (12) to 62.0% (13). Our result consists of those studies. The presented study is first national study on the body pain among elderly Chinese. The previous studies from China are focused on the specific health conditions such as hip arthritis, knee and back pain among the elderly (14, 15). Those results cannot be used to compare directly. The different results from these studies could be attributed to differences in sample populations, sampling methods, measurements, and culture factors (3). Our results indicated that older age is associated with increased severity of pain and that females tend to experience greater pain, these findings are in accordance with other studies from around the world (16–18). Cancer, osteoarthritis and rheumatoid arthritis, operations and injuries, and spinal problems are the main causes of pain (2). With aging, those chronic diseases are increased which could partly explain the aging increasing on pain prevalence. Regarding the gender difference, one possible explanation is the women might be more sensitive to pain feeling. Previous study demonstrated a significant gender-related difference in Laser-Evoked amplitudes with lower mean values in men (19).

Our study found that agricultural work was related to increased mild pain. The results are consistent with the study on Latino farmers, which showed that the odds ratio of chronic pain were higher among agricultural workers in men (OR 2.49, 95%CI: 1.03–5.99) and in women (OR 2.15, 95%CI: 1.04–4.46) (20). We also found that living in urban areas was associated with fewer odds ratio of both mild and severe pain; it is possibly due to more access to hospitals, clinics and medication in urban areas. This was in line with the study on women in rural Tibet (21), showing that the odds ratio of a low back pain was 0.34 (95% CI 0.28–0.40). Another important factor is the health condition, known to increase the chances of pain symptoms in our study (22). Participants who had chronic pain significant more often reported when they had a chronic disease (23). Regarding the association between social-economic (education level) and pain, our results are inlined which indicated an inverse correlation between socioeconomic status and chronic pain between older African American and Caucasian Americans (24). The study indicated a reverse correlation between education level and severe pain, which is in accordance with a German study where they found that educational level was inversely associated with back pain and severe current back pain (25). Many factors may have contributed to the association, such as language, literacy and access to educational resources (26). In addition, lower SES individuals may wait longer to present for their treatment, related to health system delays, physician bias, patient education or patient preference (27).

The strengths of this study include the large sample size and the adjustment for important confounders (age, sex, occupations, etc.). We also measure the intensity of the body pain, which can provide the effect information for pain management in the future. Our study did have several limitations. Pain data is self-reported which might be imprecise and subject to reporting bias. The precise objective pain measurement is warranted or the validation study on the self-reported pain issue is needed in the future study. Last, the data on the other risk factors related to pain (e.g. BMI (28), life events, psychological distress, physical activity, and medication) is lacked. Future studies with those risk factors are warranted.

Our study has special public health implication, for example, our study could provide the base to create more effective programs targeted for specific pain management for elderly Chinese. Social inequalities had effect on the body pain. Public health policies subsidize multidisciplinary pain management programs (29). However, proper pain management in China is still an ongoing problem. The main barriers to proper pain management are lack of knowledge regarding how to care for individuals with pain (30).

## Conclusion

Older age is associated with increased severity of pain and that females tend to experience greater pain. Socioeconomic variables such as education, occupation and health conditions are associated with moderate pain and severe pain.

## Ethical considerations

The study was carried out in compliance with the Declaration of Helsinki of the World Medical Association. The objectives of the study were explained to the study participants and verbal consent was obtained before interviewing each participant.
